# First Fossil Record of *Alphonsea* Hk. f. & T. (Annonaceae) from the Late Oligocene Sediments of Assam, India and Comments on Its Phytogeography

**DOI:** 10.1371/journal.pone.0053177

**Published:** 2013-01-22

**Authors:** Gaurav Srivastava, Rakesh C. Mehrotra

**Affiliations:** Birbal Sahni Institute of Palaeobotany, Lucknow, India; University of Delhi, India

## Abstract

A new fossil leaf impression of *Alphonsea* Hk. f. & T. of the family Annonaceae is described from the Late Oligocene sediments of Makum Coalfield, Assam, India. This is the first authentic record of the fossil of *Alphonsea* from the Tertiary rocks of South Asia. The Late Oligocene was the time of the last significant globally warm climate and the fossil locality was at 10°–15°N palaeolatitude. The known palaeoflora and sedimentological studies indicate a fluvio-marine deltaic environment with a mosaic of mangrove, fluvial, mire and lacustrine depositional environments. During the depositional period the suturing between the Indian and Eurasian plates was not complete to facilitate the plant migration. The suturing was over by the end of the Late Oligocene/beginning of Early Miocene resulting in the migration of the genus to Southeast Asia where it is growing profusely at present. The present study is in congruence with the earlier published palaeofloral and molecular phylogenetic data. The study also suggests that the Indian plate was not only a biotic ferry during its northward voyage from Gondwana to Asia but also a place for the origin of several plant taxa.

## Introduction

Annonaceae is a typical pantropical family of shrubs, trees and lianas consisting of about 112 genera and 2440 species [1] and is considered as one of the most diverse families of the magnoliid clade. Based on the molecular data the family has been placed in Magnoliales, along with Degeneriaceae, Eupomatiaceae, Himantandraceae, Magnoliaceae and Myristicaceae [2]. On the basis of DNA sequences [3], [4] and other morphological features [5] the family is strongly considered as monophyletic in origin. It has been considered as an important component of lowland tropical rainforest around the world [6]–[9], whose abundance and species richness covary with the temperature and rainfall [9].

**Figure 1:**
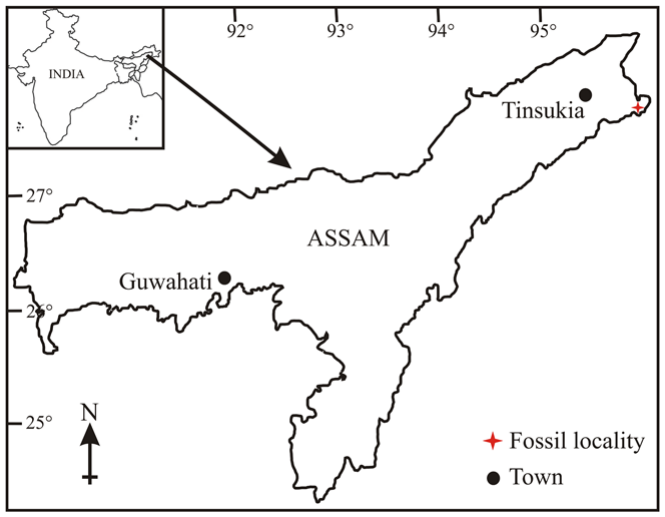
Map showing the fossil locality (red astrick).

The oldest fossil records of Annonaceae are in the form of seeds and pollen from the Maastrichtian of Nigeria and Colombia [10], [11] indicating that it is Gondwanic in origin despite the fact that one of the fossil records is from the Coniacean (Late Cretaceous) of Japan [12]. Fossil records of Annonaceae are well documented in the Neogene in contrast to the Palaeogene sediments of India [13]–[15]. In the present paper we describe a new leaf impression of *Alphonsea* from the Late Oligocene (Chattian 28–23 Ma) sediments of Makum Coalfield, Assam (Fig. 1), which was located at 10°–15°N palaeolatitude during the depositional period [16] when the suturing of the Indian and Eurasian plates was not complete to facilitate the plant migration [17]. The known palaeoflora and sedimentological studies indicate a fluvio-marine deltaic environment with a mosaic of mangrove, fluvial, mire and lacustrine depositional environments. [18], [19]. This is the first fossil record of the genus *Alphonsea* from the Tertiary rocks of South Asia. An attempt is also made to discuss the evolution and migration of the genus in the region.

### Geological setting

**Figure 2:**
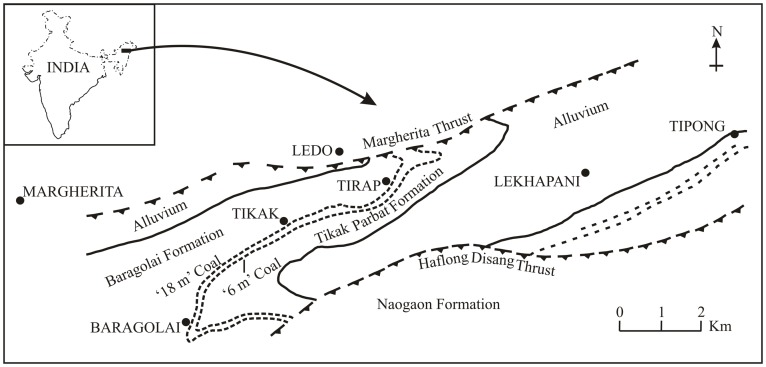
Simplified geological map of the Makum Coalfield, Assam [**52**].

Of the several coalfields in northeast India the Makum Coalfield is the most important as it accounts for nearly 90% coal production in this part of the country. This field lies between the latitudes 27°15′–27°25′N and longitudes 95°40′–95°55′E (Fig. 1) and is situated along the northern flank of the Patkai range. On the southern and south-eastern side of the field are hills, which rise abruptly to heights of 300–500 m from the alluvial plains of the Buri Dihing and Tirap rivers. These hill ranges are traversed by the Namdang, Ledopani and Tirap rivers. These river courses expose sections of the coalbearing Tikak Parbat Formation. The Makum Coalfield encompasses Baragolai, Ledo, Namdang, Tikak, Tipong, and Tirap collieries (Fig. 2).

The present study is concerned with the Tikak Parbat Formation assigned to Late Oligocene (Chattian 28–23 Myr) [18], [19]. The formation has five coal seams confined within the basal 200 m section [20].

Of the five seams only seam no. 1 and 3 have been exploited throughout the field. The Tikak Parbat Formation comprises alternations of sandstone, siltstone, mudstone, shale, carbonaceous shale, clay and coal seams [20] (Fig. 2). However, the occurrence of plant remains is mainly confined to the grey carbonaceous and sandy shales. The formation is underlain by 300 m of predominantly massive, micaceous or ferruginous sandstones that comprise the Baragolai Formation, which in turn is underlain by 1100–1700 m of thin-bedded fine-grained quartzitic sandstones with thin shale and sandy shale partings that make up the Naogaon Formation [21]. Together the three formations comprise the Barail Group (Fig. 2). In the Barail Group there is an upward trend from predominantly marine to predominantly non-marine palaeoenvironments which represent the infilling of a linear basin on the eastern edge of the Indian plate. The detailed geological account of the Tirap mine section has recently been published [18].

## Materials and Methods

Material for the present study was collected from the Tirap Colliery (27°17′20″N, 95°46′15″E) of the Makum Coalfield, Tinsukia District, Assam having an exposure of the Late Oligocene sediments belonging to the Tikak Parbat Formation of Assam. The specimen was first cleared with the help of a fine chisel and hammer and then photographed in natural low angled light using 10 megapixel digital camera (Canon SX110 and Fuji color 9500).The terminology used in describing the fossil leaf is based on Hickey [22], Dilcher [23] and Ellis et al. [24]. The type specimen is housed in the museum of the Birbal Sahni Institute of Palaeobotany, Lucknow, bearing specimen no. BSIP 40084

### Nomenclature

The electronic version of this article in Portable Document Format (PDF) in a work with an ISSN or ISBN will represent a published work according to the International Code of Nomenclature for algae, fungi, and plants, and hence the new names contained in the electronic publication of a PLOS ONE article are effectively published under that Code from the electronic edition alone, so there is no longer any need to provide printed copies. The online version of this work is archived and available from the following digital repositories: PubMed Central, LOCKSS.

## Results

**Family.** Annonaceae Juss.

**Genus.** *Alphonsea* Hk. f. & T.

**Species.** *A. makumensis* Srivastava & Mehrotra, sp. nov.

**Figure 3:**
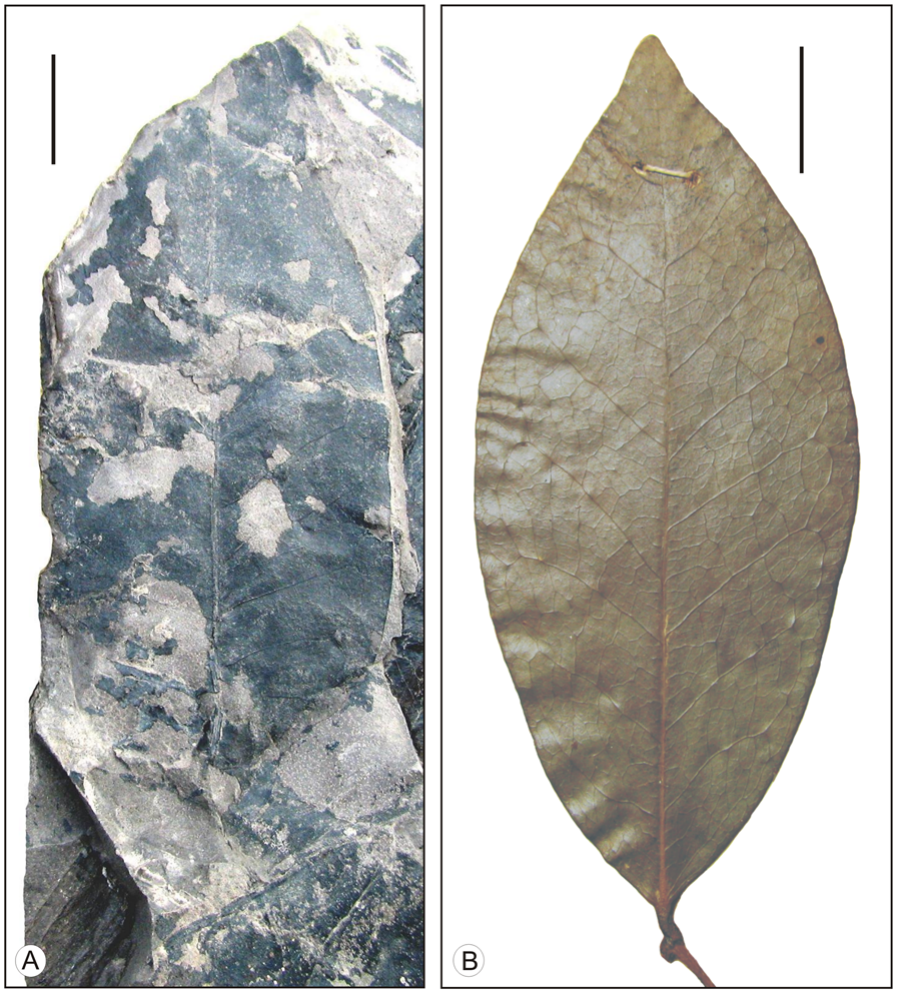
*Alphonsea* leaves. A. Fossil leaf of *A. makumensis* sp. nov. showing shape, size and venation pattern. B. Modern leaf of *A. lutea* showing similar shape, size and venation pattern (Scale bar = 1 cm).

**Figure 4:**
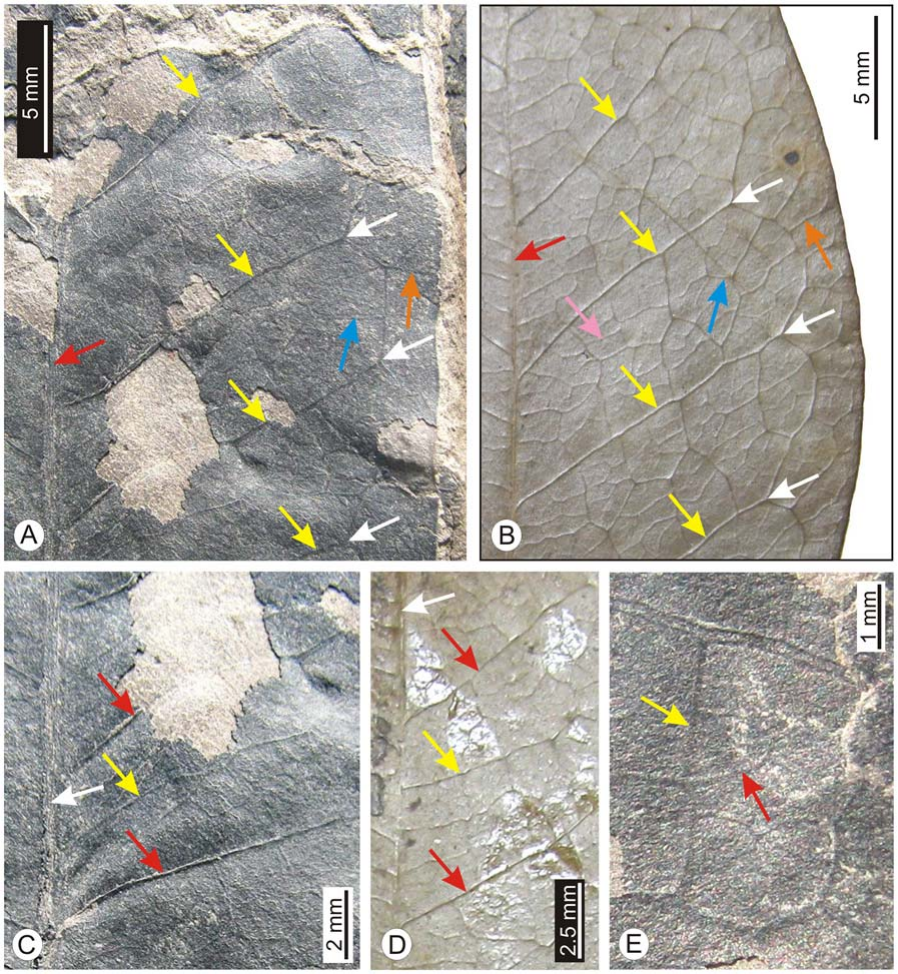
*Alphonsea* leaves. A. Enlarged portion of the fossil leaf showing primary vein (red arrow), secondary veins (yellow arrows), brochidodromous venation (white arrows), random reticulate tertiary vein (blue arrow) and exmedial tertiary vein (orange arrow). B. Enlarged portion of the modern leaf of *A. lutea* showing similar primary vein (red arrow), secondary veins (yellow arrows), brochidodromous venation (white arrows), random reticulate tertiary vein (blue arrow), exmedial tertiary vein (orange arrow) and quadrangular areole (pink arrow). C. Enlarged portion of the fossil leaf showing primary vein (white arrow), secondary veins (red arrows) and intersecondary vein (yellow arrow). D. Modern leaf of *A. lutea* showing similar primary vein (white arrow), secondary vein (red arrows) and intersecondary vein (yellow arrow). E. Enlarged portion of the fossil leaf showing single quadrangular areole (yellow arrow) and free veinlets (red arrow).

**Figures** 3A; 4A,C,E

**Holotype.** Specimen no. BSIP 40084

**Horizon.** Tikak Parbat Formation, Barail Group

**Locality.** Tirap Colliery (27°17′20″N, 95°46′15″E), Tinsukia District, Assam

**Age.** Late Oligocene (28–23 Ma)

**Number of specimens studied.** One

### Diagnosis

Leaf elliptic-narrow elliptic; margin entire; venation festooned brochidodromous; secondary veins 0.3–1.3 cm apart, angle of divergence moderate to wide acute; intersecondary veins present; tertiary veins random reticulate; marginal ultimate venation looped.

#### Description

Leaf complete, symmetrical, mesophyll, elliptic-narrow elliptic; preserved lamina length 7.6 cm, maximum width 3.2 cm (near the middle portion); apex broken; base symmetrical, seemingly obtuse; margin entire; texture coriaceous; attachment with petiole normal, petiole 0.7 cm long; venation festooned brochidodromous; primary vein moderate in thickness, straight; secondary veins 12 pairs visible, 0.3–1.3 cm apart, not uniform, alternate to sub-opposite, angle of divergence moderate-wide acute (45°–69°), moderate in thickness, joining super-adjacent secondary veins at acute-obtuse angle; intersecondary veins present, composite; tertiary veins random reticulate; marginal ultimate venation looped; areoles present, quadrangular to pentagonal in shape; veinlets branched.

#### Affinities

The characteristic features of the fossil leaf viz., elliptic-narrow elliptic shape, coriaceous texture, festooned brochidodromous venation, intersecondary veins and random reticulate tertiary veins suggest its close affinity with that of *Alphonsea* of the family Annonaceae. A number of species of *Alphonsea*, namely *A. lutea* Hk. f. & Th., *A. madraspertana* Bedd., *A. sclerocarpa* Thw. and *A. ventricosa* Hk. f. & Th. along with other taxa of the same family examined in the Central National Herbarium, Howrah, India and the Forest Research Institute, Dehradun, India. In *A. sclerocarpa* the distance between the two secondary veins is greater than that found in the present fossil, while in *A. ventricosa* the venation is eucamptodromous. *A. lutea* (Herberium sheet no. CNH 12209) and *A. madraspertana* (Herbarium sheet no. CNH 14643) are very similar to the fossil leaf (Fig. 3B; 4B,D). The fossil also shows some resemblance with *Annona senegalensis* Pers., *Uvaria hookeri* King and *Uvaria zeylanica* L. of the same family but the presence of eucamptodromous-brochidodromous venation makes the difference from the present fossil.

**Figure 5:**
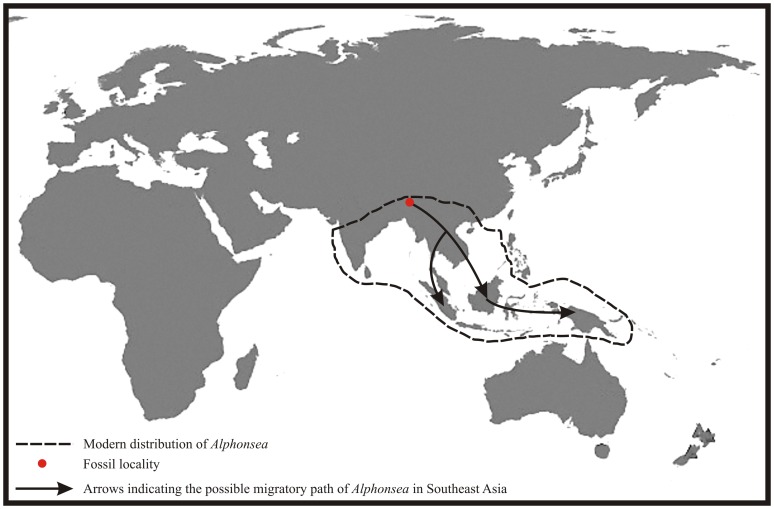
Showing modern distribution of *Alphonsea* and possible migratory path of *Alphonsea* in Southeast Asia.

Hably [25] reported *Alphonsea* fossil from the Miocene sediments of Hungary but the modern distribution of the genus being restricted only to South and Southeast Asia (Fig. 5) creates doubt on the Hungarian fossil. Under such circumstances a new species, *Alphonsea makumensis* Srivastava & Mehrotra, sp. nov., is created, the specific epithet is after the Makum Coalfield. As far as authors are aware, this is the first authentic fossil leaf record of *Alphonsea* from Southeast Asia.

### Modern distribution of *Alphonsea*

The genus *Alphonsea* consists of about 30 species distributed in China and Indo-Malayan region [26] (Fig. 5). In China it is mainly found in Hainan and South Yunnan [27], while in Asia it is found in India, Sri Lanka, Myanmar, Thailand, Laos, Vietnam, Cambodia, Malaysia, Indonesia and Papua New Guinea [28]. One of the modern comparable species, *Alphonsea lutea*, is a tree of Bangladesh, Myanmar, Orissa and Sri Lanka, while the other one, *A. madraspertana*, is an evergreen tree of the hills of Cuddapah District, Andhra Pradesh and Thiruvannaamalai District, Tamil Nadu [29].

## Discussion

On the basis of molecular phylogenetic study within the Annonaceae, two major sister clades were recognized containing the majority of the species, namely the long-branch clade (LBC) and short-branch clade (SBC) [1], [30]. The genus *Alphonsea* belongs to SBC of the tribe Miliuseae which comprises of the following six genera distributed in Asia: *Alphonsea*, *Mezzettia* Becc., *Miliusa* Lesch. ex A.DC., *Orophea* Bl., *Phoenicanthus* Alston and *Platymitra* Boerl. Couvreur et al. [1] suggested that the age of the crown node of SBC is c.33 Ma. Other molecular data suggests that the *Alphonsea* was separated from its sister genus *Platymitra* during the Late Oligocene [30]; this is in contrast to Couvreur et al. [1] data which suggests that the separation occurred during the Late Miocene. Our leaf fossil is in favour of Richardson's et al. [30]. Su and Saunders [31] suggested the role of rafting Indian plate in the dispersal of SBC into Southeast Asia i.e. “Out of India hypothesis”. Their idea gets more support from the fossil record of the family Annonaceae from the Deccan Intertrappean sediments [32]–[36] suggesting that the family was already present before the collision of Indian and Eurasian plates.

During the Late Oligocene the fossil locality was at 10°–15°N [16] and the suturing between the Indian and Eurasian plates was not complete to facilitate the plant migration [17]. The presence of *Alphonsea* fossil in northeast India during the Late Oligocene indicates that the genus was originated in India during the Late Oligocene and migrated to Southeast Asia via Myanmar after the complete suturing of Indian and Eurasian plates during the Early Miocene [37]. There are several examples which support that a significant floral exchange happened between India and Southeast Asia during the Neogene. Sterculiaceae is an interesting example as it is represented in the Palaeogene of Southeast Asia by *Pterospermum* and in India by *Sterculia*. The former made its first appearance in the Pliocene [38], while the latter is known from the Neogene of Sumatra [39]. Similar is the case with the *Mangifera* and *Semecarpus* of the family Anacardiaceae. The oldest fossil records of these taxa are known from the Palaeogene of India. These genera later on migrated to Southeast Asia during the Neogene as evidenced by their fossil records [40]–[42]. The other plant families like Crypteroniaceae [43], [44], Melastomataceae [45] and Lowiaceae [46], along with the palaeotological evidence [47] support the above fact as they were migrated from India to Southeast Asia. The present study also suggests that the Indian plate was not only a biotic ferry during its northward voyage from Gondwana to Asia but also a place for the origin of several plant taxa.

For the reconstruction of palaeoclimate of the Makum Coalfield both qualitative and quantitative studies have been made. Qualitative study is based on the floral composition of the families such as Annonaceae, Burseraceae, Calophyllaceae, Combretaceae, Lecythidaceae, Myristicaceae and Rhizophoraceae. The aforesaid families are typical pantropical [48] and their presence in the Makum Coalfield palaeoflora provides evidence that the CMMT (cold month mean temperature) was not less than 18°C. Fabaceae, the most dominant family in the Makum Coalfield [49] whose abundance and richness covary with temperature [9], also indicates a warm climate. The occurrence of Avicenniaceae and Rhizophoraceae is significant in terms of the depositional environment. These families are highly indicative of deltaic, mangrove or lacustrine deposition of sediments in the Makum Coalfield. The presence of palms like *Nypa* [50] provides further evidence of a coastal plain environment where both temperature and humidity remained high throughout the year [51]. For quantitative study, CLAMP (Climate Leaf Analysis Multivariate Program) analysis was made indicating MAT (mean annual temperature) 28.3±3.7°C, CMMT (cold month mean temperature) 23±5.5°C and a WMMT (warm month mean temperature) of 33.6±5.2°C. The analysis also indicates a monsoonal climate during the Late Oligocene [19].

## References

[1] Couvreur TLP, Forest F, Baker WJ (2011) Early evolutionary history of the flowering plant family Annonaceae: steady diversification and boreotropical geodispersal. J Biogeogr 38: 664–680.

[2] APG II (2003) An update of the Angiosperm Phylogeny Group classification for the orders and families of flowering plants: APG II. Bot J Linn Soc 141: 399–436.

[3] Qiu Y-L, Chase MW, Les DH, Parks CR (1993) Molecular phylogenetics of the Magnoliidae: cladistic analyses of nucleotide sequences of the plastid gene *rbcL* . Ann Missouri Bot Gard 80: 587–606.

[4] Qiu Yin-Long, Lee J, Bernasconi-Quadroni F, Soltis DE, Soltis PS, et al (1999) The earliest angiosperms: evidence from mitochondria plastid and nuclear genomes. Nature 402: 404–407. 10586879 10.1038/46536

[5] Doyle JA, Thomas ALe (1994) Cladistic analysis and pollen evolution in Annonaceae. Acta Bot Gall 141: 149–170.

[6] Gentry A (1993) Diversity and floristic composition of lowland tropical forest in Africa and South America. In: Goldblatt P, ed. Biological relationships between Africa and South America. New Haven: Yale University Press. pp 500–547.

[7] Burnham RJ, Johnson KR (2004) South American palaeobotany and the origins of neotropical rainforests. Phil Trans R Soc Lond B 359: 1595–1610. 15519975 10.1098/rstb.2004.1531 PMC1693437

[8] Tchouto MGP, de Boer WF, de Wilde JJFE, van der Maesen LJG (2006) Diversity patterns in the flora of the Campo-Ma'an rain forest, Cameroon: do tree species tell it all? Biodiversity Conserv 15: 1353–1374.

[9] Punyasena SW, Eshel G, McElwain JC (2008) The influence of climate on the spatial patterning of Neotropical plant families. J Biogeogr 35: 117–130.

[10] Chesters KIM (1955) Some plant remains from the Upper Cretaceous and Tertiary of West Africa. Ann Mag Nat Hist 8 12: 498–504.

[11] Sole de Porta N (1971) Algunos generos nuevos de polen procedentes de la formacion Guaduas (Maastrichtiense-Paleocene) de Colombia. Stud Geol Salamanca 2: 133–143.

[12] Takahashi M, Friis EM, Uesugi K, Suzuki Y, Crane PR (2008) Floral evidence of Annonaceae from the Late Cretaceous of Japan. Int J Plant Sci 169 7 908–917.

[13] Lakhanpal RN, Maheshwari HK, Awasthi N (1976) A catalogue of Indian fossil plants. Lucknow: Birbal Sahni Institute of Palaeobotany. pp 1–318.

[14] Srivastava R (1991) A catalogue of fossil plants from India–4. Cenozoic (Tertiary) megafossils. Lucknow: Birbal Sahni Institute of Palaeobotany. pp 1–45.

[15] Srivastava R, Guleria JS (2005) A catalogue of Cenozoic (Tertiary) plant megafossils from India (1989–2005). Lucknow: Birbal Sahni Institute of Palaeobotany. pp 1–76.

[16] Molnar P, Stock JM (2009) Slowing of India's convergence with Eurasia since 20 Ma and its implications for Tibetan mantle dynamics. Tectonics 28: TC3002.

[17] Srivastava G, Mehrotra RC (2010) Tertiary flora of northeast India vis-à-vis movement of the Indian plate. Mem Geol Soc India 75: 123–130.

[18] Kumar M, Srivastava G, Spicer RA, Spicer TEV, Mehrotra RC, et al (2012) Sedimentology, palynostratigraphy and palynofacies of the Late Oligocene Makum Coalfield, Assam, India: a window on lowland tropical vegetation during the most recent episode of significant global warmth. Palaeogeogr Palaeoclimatol Palaeoecol 342–343: 143–162.

[19] Srivastava G, Spicer RA, Spicer TEV, Yang J, Kumar M, et al (2012) Megaflora and palaeoclimate of a Late Oligocene tropical delta, Makum Coalfield, Assam: evidence for the early development of the South Asia monsoon. Palaeogeogr Palaeoclimatol Palaeoecol 342–343: 130–142.

[20] Misra BK (1992) Tertiary coals of Makum Coalfield, Assam, India; petrography, genesis and sedimentation. Palaeobotanist 39 3 309–326.

[21] Mishra HK, Ghosh RK (1996) Geology, petrology and utilization of some Tertiary coals of the northeastern region of India. Int J Coal Geology 30: 65–100.

[22] Hickey LJ (1973) Classification of the architecture of dicotyledonous leaves. Am J Bot 60: 17–33.

[23] Dilcher DL (1974) Approaches to the identification of angiosperm leaf remains. Bot Rev 40: 1–157.

[24] Ellis B, Daly DC, Hickey LJ, Johnson KR, Mitchell JD, et al. (2009) Manual of leaf architecture. USA: Cornell University Press.

[25] Hably L (2006) Catalogue of the Hungarian Cenozoic leaf, fruit and seed floras from 1856 to 2005. Studia Bot Hung 37: 41–129.

[26] Mabberley DJ (1997) The plant book, a portable dictionary of the vascular plants. Cambridge: Cambridge University Press.

[27] Flora of China Website. http://www.efloras.org/florataxon.aspx?flora_id=2&taxon_id=101186. Accessed 2012 Aug 10.

[28] Global Biodiversity Information Facility Website. http://data.gbif.org/species/3154131/. Accessed 2012 Aug 10.

[29] Gamble JS (1972) A manual of Indian timbers. Dehradun: Bishen Singh Mahendra Pal Singh.

[30] Richardson JE, Chatrou LW, Mols JB, Erkens RHJ, Pirie MD (2004) Historical biogeography of two cosmopolitan families of flowering plants: Annonaceae and Rhamnaceae. Phil Trans R Soc B 359: 1495–1508. 15519968 10.1098/rstb.2004.1537 PMC1693429

[31] Su YCF, Saunders RMK (2009) Evolutionary divergence times in the Annonaceae: evidence of a Late Miocene origin of *Pseuduvaria* in Sundaland with subsequent diversification in New Guinea. BMC Evol Biol 9: 153. 19573225 10.1186/1471-2148-9-153 PMC2722625

[32] Carter HJ (1852) Summary of the geology of India, between the Ganges, the Indua and cape Comorin. J Bombay Brch R Asiat Soc 5: 179 (reprinted in Geoloical papers on western India, Carter HJ, ed., 1857. Bombay: Education Soc Press. pp 628–776.

[33] Bande MB (1973) A petrified dicotyledonous wood from the Deccan Intertrappean beds of Mandla District, Madhya Pradesh. Botanique 4 1: 41–47.

[34] Mehrotra RC (1990) Further observations on some fossil woods from the Deccan Intertrappean beds of Central India. Phytomorphology 40 1–2: 169–174.

[35] Bonde SD (1993) *Unonaspermum corneri* gen. et sp. nov., an annonaceous seed from the Deccan Intertrappean beds of India. J Indian Bot Soc 72: 251–253.

[36] Guleria JS, Mehrotra RC (1999) On some plant remains from Deccan Intertrappean localities of Seoni and Mandla districts of Madhya Pradesh, India. Palaeobotanist 47: 68–87.

[37] Chatterjee S, Scotese CR (1999) The breakup of Gondwana and the evolution and biogeography of the Indian plate. Proc Indian Nat Sci Acad 65A: 397–425.

[38] Lakhanpal RN, Guleria JS (1983) A preliminary appraisal of the Tertiary megaflora of Kachchh District, Gujarat, western India. Geophytology 13: 46–54.

[39] Bande MB, Prakash U (1986) The Tertiary flora of Southeast Asia with remarks on its palaeoenvironment and phytogeography of the Indo-Malayan region. Rev Palaeobot Palynol 49: 203–233.

[40] Mehrotra RC, Dilcher DL, Awasthi N (1998) A Palaeocene *Mangifera*- like leaf fossil from India. Phytomorphology 48: 91–100.

[41] Mehrotra RC (2000) Study of plant megafossils from the Tura Formation of Nangwalbibra, Garo Hills, Meghalaya. Palaeobotanist 49: 225–237.

[42] Srivastava G, Mehrotra RC (2012) Oldest fossil of *Semecarpus* L.f. from the Makum Coalfield, Assam, India and comments on its origin. Curr Sci 102 3: 398–400.

[43] Conti E, Eriksson T, Schönenberger J, Sytsma KJ, Baum DA (2002) Early Tertiary Out-of-India dispersal of the Crypteroniaceae: evidence from phylogeny and molecular dating. Evolution 56: 1931–1942. 12449480 10.1554/0014-3820(2002)056[1931:ETOOID]2.0.CO;2

[44] Rutschmann F, Eriksson T, Schönenberger J, Conti E (2004) Did Crypteroniaceae really disperse out of India? Molecular dating evidence from rbcL, ndhF, and rpl16 intron sequences. Int J Plant Sci 165: S69–S83.

[45] Morley RJ, Dick CW (2003) Missing fossils, molecular clocks, and the origin of the Melastomataceae. Am J Bot 90: 1638–1644. 21653339 10.3732/ajb.90.11.1638

[46] Kress JW, Specht CD (2006) The evolutionary and biogeographic origin of the tropical monocot order Zingiberales. Aliso 22: 619–630.

[47] Bossuyt F, Milinkovitch MC (2001) Amphibians as indicators of early Tertiary “Out-of-India” dispersal of vertebrates. Science 292: 93–95. 11292870 10.1126/science.1058875

[48] von Steenis CGGJ (1962) The land-bridge theory in botany. Blumea 11 1: 235–372.

[49] Srivastava G, Mehrotra RC (2010) New legume fruits from the Oligocene sediments of Assam. J Geol Soc India 75: 820–828.

[50] Mehrotra RC, Tiwari RP, Mazumder BI (2003) *Nypa* megafossils from the Tertiary sediments of northeast India. Geobios 36: 83–92.

[51] Tomlinson PB (1990) The structural biology of palms. Oxford: Clarendon Press.

[52] Ahmed M (1996) Petrology of Oligocene coal, Makum coalfield, Assam, northeast India. Int J Coal Geol 30: 319–325.

